# Visual Field Defect after Cardiac Surgery: The Striking Role of Interdisciplinary Collaboration

**DOI:** 10.1155/2015/904528

**Published:** 2015-12-07

**Authors:** Raffaele Nuzzi, Carlo Lavia

**Affiliations:** Dipartimento Di Scienze Chirurgiche, Clinica Oculistica Dell'Università, Via Juvarra 19, 10122 Turin, Italy

## Abstract

Perioperative visual loss (POVL) is a potentially devastating complication that can occur following ocular or nonocular surgery. The leading causes of this disease are retinal vascular occlusions, ischemic optic neuropathies, and cortical blindness. POVL pathogenesis is strictly influenced by surgery, anesthesia, and patients' comorbidities. We report of a 55-year-old caucasian man who presented with complaints of sudden painless loss of vision and unilateral campimetric deficit. We recorded a preserved visual acuity but at fundus examination a bilateral ischemic optic neuropathy (ION) was suspected. Our hypothesis was supported by uncommon and peculiar visual field defects and a history of cardiovascular surgery shortly before was a striking data. When we examined his medical records we found strong accordance with what is reported in literature to be risk factors for postoperative ION development. He presented intraoperative hypotension, anemia, and hypothermia, he was older than 50 years, and surgery lasted for more than five hours. We are currently monitoring his visual acuity and visual fields which remain unchanged. As there is no proved therapy for such severe adverse events, we recommend intraoperative check of blood pressure, blood loss, and body temperature, associated with repeated eye checks and patients' interview.

## 1. Introduction

Visual loss is a rare but potentially devastating complication that can be seen after ocular or nonocular surgery. It can be either transient or permanent and can affect one or more ocular structures, from the cornea to the optic nerve.

In some cases visual loss is permanent and severe and it is known as perioperative visual loss (POVL). In a systematic review of more than 5 million patients who underwent cardiac, spinal, orthopedic, or general surgery in the United States, the prevalence was 0.0235% [1].

The most common causes of POVL include retinal vascular occlusions (65.16%), ischemic optic neuropathies (ION) (18.48%), and cortical blindness (16.21%) [1].

The type of surgery and anesthesia performed, the comorbidities of the patient, the intraoperative conditions, and any surgical complication may play an essential role in the pathogenesis of POVL.

## 2. Case Report

A 55-year-old caucasian man appeared to our center with complaints of sudden painless loss of vision of recent onset (about a month) and campimetric deficit in the lower fields of his right eye. His ophthalmologic medical history was negative till that moment.

On presentation the patient had a best corrected visual acuity of 20/25 and a corrected near vision of J3 (Jaeger chart) in both eyes. Slit lamp examination showed unaltered anterior segment with no evidence of inflammation and an intraocular pressure (IOP) of 16 mmHg. Direct and consensual pupillary reflexes were present but slightly sluggish with a relative afferent pupil defect in the right eye. Dilated-pupil fundus examination in both eyes showed a round and pale optic disc with defined margins; macular region and retinal vessels were normal.

**Figure 1:**
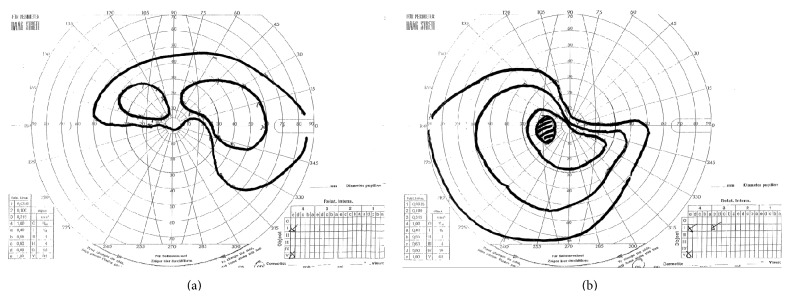
Goldmann perimetry. (a) Right eye, (b) left eye.

**Figure 2:**
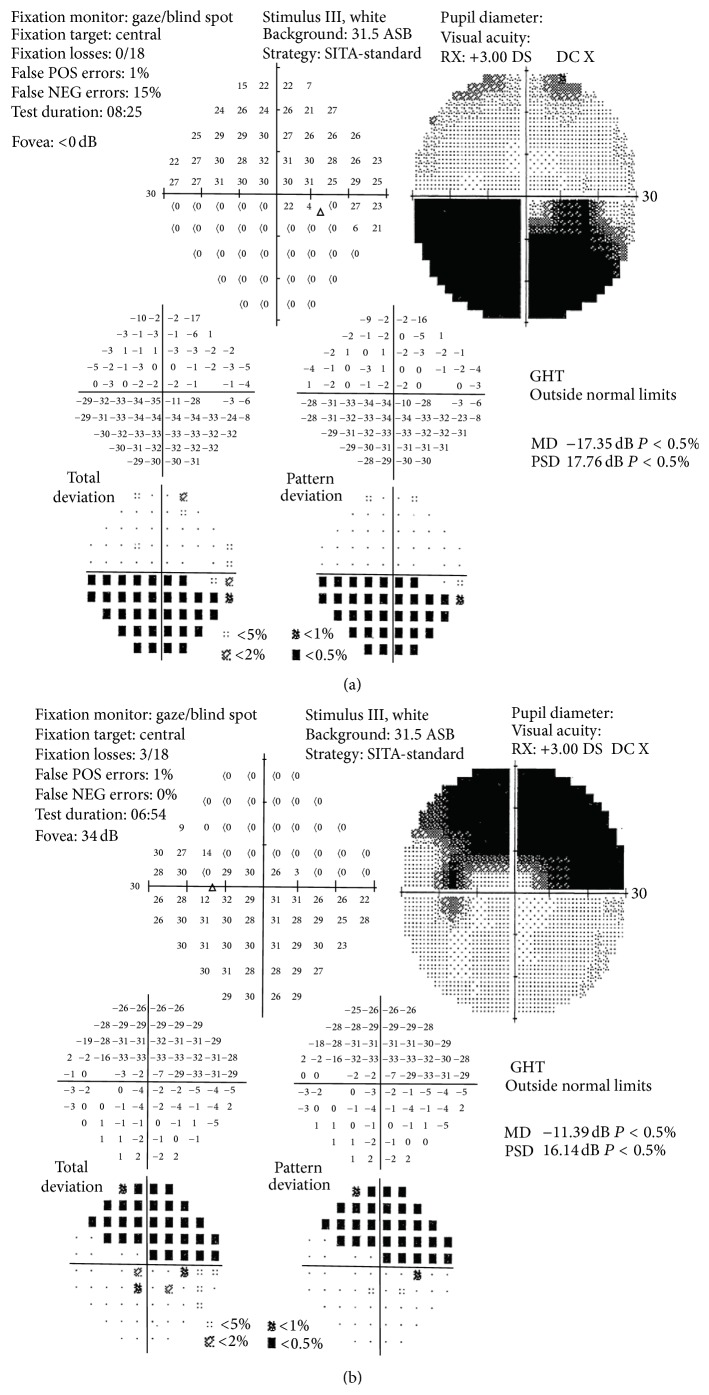
Humphrey visual field. (a) Right eye, (b) left eye.

Hess test and Lang stereotest were both performed and the results were normal. Goldmann perimetry showed an altitudinal deficit in both eyes, involving opposite hemifields (Figure 1) confirmed at automated perimetry (Humphrey 30-2 SITA-standard, Carl Zeiss Meditec, Dublin, CA) (Figure 2).

During the first visit we performed a laboratory evaluation including complete blood count, erythrocyte sedimentation rate (ESR), and C-reactive protein (CRP), considering other potential causes of optic neuropathy, and we prescribed oral prednisone 25 mg per day. All the blood exams made were within the normal range.

His family history was positive for cardiovascular diseases (brother with heart attack) and he suffered from angina pectoris, hypertension, and hypercholesterolemia in therapy with atenolol, beta-blockers, and statins.

A relevant data went out as we discovered that in July 2014 (eight weeks before our first examination) he underwent a coronary bypass (4 bypasses) for a significant stenosis involving both the left coronary artery (in the main and the anterior interventricular branch) and the right coronary artery ostium.

From the examination of the medical records we observed that the total duration of surgery was 5.5 hours, including 2 hours of extracorporeal circulation (ECC). No intraoperative complication was reported and the patient was weaned without difficulties from the ECC.

**Figure 3:**
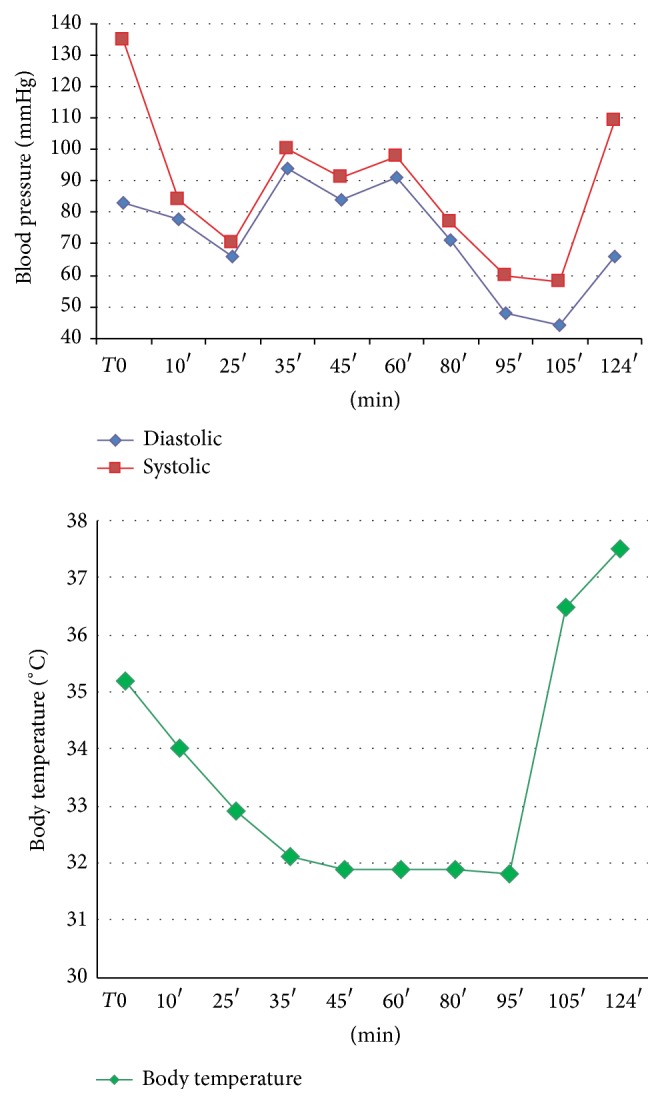
ECC body temperature and blood pressure records.

During the ECC a systolic blood pressure between 130 and 77 mmHg and a diastolic blood pressure between 94 and 44 mmHg were maintained. The mean arterial pressure (MAP) in ECC was equal to 73.17 mmHg (Figure 3).

Recorded blood pH variations were in normal range while hemoglobin (Hb) values fell from 15.4 g/dL (hematocrit (Ht) 47.2%) at the beginning to 9.4 g/dL (Ht 29.5%) at the end of the ECC.

During the period of cardioplegia the blood flow was maintained between 4000 and 5520 cc/min and patient's body temperature (Figure 3) went down to 32°C after 30 minutes from the beginning of the ECC and went back within normal values at its conclusion.

The postoperative course was characterized by low-grade fever (negative blood cultures) and psychomotor agitation, which resolved within 72 hours.

In the second postoperative day it was necessary to transfuse 2 bags of packed red blood cells due to persistently low Hb values: 8.5 g/dL (Ht: 26.5%).

Upon awakening the patient complained of painless vision loss and a campimetric deficit in his right eye. After the hemodynamic parameters had been stabilized and the cardiopulmonary rehabilitation interventions had been set, our patient underwent a neurological consultation (no observable signs of recent neurological events) and a cranial computed tomography (CT) scan with contrast dye, which showed an abnormal impregnation of the globus pallidus (probably due to chronic hypoxia) but no signs of recent ischemic damage, confirmed at the Magnetic Resonance Imaging (MRI) with contrast dye.

When he came back to our clinics two weeks, 1 month, and 3 months after our first visit we did not observe significant variations: visual acuity, slit-lamp examination, and visual fields were unchanged.

At the one-month visit we decided to discontinue the steroids we prescribed during the first visit (prednisone 25 mg quaque die).

The patient is currently visited by the team of cardiologists for routine visits: the cardiovascular compensation is satisfactory and he is asymptomatic even under stress.

## 3. Discussion

POVL is a rare and potentially devastating event and ischemic optic neuropathies (ION) are among its leading causes.

ION can be classified as Anterior (AION) or Posterior (PION), depending on which part of the optic nerve has been affected, respectively, anterior or posterior to the lamina cribrosa, a semi-rigid piece of connective tissue through which the optic nerve and the central retinal vessels pass.

Postoperative ION presentation includes sudden painless vision loss at variable times of onset, between waking-up and a few days after surgery. Visual loss from PION is usually more severe than that from AION, but typically it does not worsen after its presentation. Pupil examination often reveals poorly reactive pupils and a relative afferent pupillary defect. A binocular involvement may occur in 66% of the cases [2].

Fundus examination in the acute phase is able to distinguish between the two forms: in case of AION there is a swollen optic disc with or without flame-shaped peripapillary hemorrhages, while in case of PION it seems normal.

After 4–6 weeks, both forms are characterized by optic nerve pallor of different entity.

Visual field deficit is variable but more often altitudinal or scotomatous (central or total).

Even if the pathogenesis of perioperative ION remains undefined, a higher incidence has been recorded in case of great perioperative blood loss (>1 liter) [2], hypothermia, hypotension (MAP < 70 mmHg, systolic pressure < 90 mmHg) [3], surgery lasting more than 5 hours, and patients older than 50 [1].

Lee et al. [2] found that 94% of ION cases after spine surgery had an anesthesia lasting more than 5 hours, and in 82% of them there was a blood loss which was greater than one liter. Perioperative anemia (Hb < 10 g/dL or Ht < 30%) [3], with or without hypotension, stimulates the release of endogenous vasoconstrictors due to the activation of the sympathetic nervous system, causing choroid and optic nerve ischemia.

Hypothermia, inducing an increase in blood viscosity, determines a reduction of cerebral blood flow by 6-7% for every Centigrade degree of reduction in body temperature [4].

Also perioperative hemodilution, which causes a reduction in oncotic pressure with subsequent IOP rising and interstitial edema, may play an important role in the pathogenesis of the disease.

Intraocular pressure (IOP) has documented effects on optic nerve head and its increases could promote the onset of a ION: it has been shown that there is a great IOP spike when ECC begins and that IOP can remain elevated till 72 hours from that event [5].

Our patient can be considered as an emblematic case of postoperative ION presenting the following:

1. Hypotension: MAP was below 70 mmHg in three measurements during the ECC and systolic pressure was below 90 mmHg in five measurements.
2. Anemia: Hb values were 9.4 g/dL during the ECC (Ht 29.5%) and 8.5 g/dL (Ht 26.5%) in the postoperative period (a transfusion of 2 bags of packed red blood cells was required).
3. Hypothermia: our patient experienced a moderate hypothermia (≤34°C) for a prolonged period during the ECC.
4. Age over 50 years.
5. Surgery longer than 5 hours.

As a result of the preservation of the macular region, our patient has maintained an excellent visual acuity, even if reporting an annoying visual field defect in the right eye. Although the patient reported unilateral amputation of his visual field, there was a peculiar absolute altitudinal defect, which was bilateral and mutual: probably the patient was not able to realize the presence of the defect in his left eye because of its location (the upper sectors visual field is less used and the patient already suffered from dermatochalasis) and its smaller extension.

Currently there are no treatment strategies for perioperative ION supported by the literature. High doses of steroids, correction of hypotension with vasopressors, and correction of anemia with blood transfusion have been used without consistent benefit: full visual acuity recover without any treatment is described [6], suggesting that the improvement seen after the treatments may be coincidental.

Since optic nerve blood flow impairment is related to the development of ischemic optic neuropathy, it is essential to maintain a stable ocular perfusion during surgery by elevating the MAP or decreasing the IOP.

In a recent study Molloy and Cong [7] demonstrated that perioperative Dorzolamide-Timolol drops “significantly reduce elevated IOP of patients who undergo lengthy laparoscopic, robotic surgery in the steep Trendelenburg (ST) position,” suggesting that protocols of treatment are recommended in order to obtain a level of ophthalmic safety in patients undergoing prolonged surgery in prone or ST position.

In major surgery, a strict collaboration between anesthesiologists, surgeons, and ophthalmologists is essential: continuous blood pressure control, blood loss and temperature monitoring, use of colloids to maintain euvolemia, frequent IOP measurements, repeated eye checks (especially to explore central retinal artery perfusion), and patients' interview during spine surgery are recommended.

During the preoperative interview with patients anticipated to undergo prolonged surgery with great blood loss it is essential to discuss the remote risk of POVL and, if necessary, to prescribe an adequate prophylaxis with eyedrops.

A close interdisciplinary collaboration is to be hoped in order to achieve better management of such cases that can be considered anything but just ophthalmologic diseases.
